# Only giving orders? An experimental study of the sense of agency when giving or receiving commands

**DOI:** 10.1371/journal.pone.0204027

**Published:** 2018-09-26

**Authors:** Emilie A. Caspar, Axel Cleeremans, Patrick Haggard

**Affiliations:** 1 Consciousness, Cognition and Computation Group (CO3), Center for Research in Cognition & Neurosciences (CRCN), ULB Neuroscience Institute (UNI), Université libre de Bruxelles (ULB), Brussels, Belgium; 2 Institute of Cognitive Neuroscience, University College London (UCL), London, United Kingdom; University G d'Annunzio, ITALY

## Abstract

In human societies, agents are assumed to experience being the author of their own actions. These basic motoric experiences of action are influenced by social hierarchies, leading to surprising and morally significant results. Here we ask whether, under coercion, the sense of agency and responsibility pass from the person who receives orders to the person who gives them. Volunteers took turns to play the roles of ‘commander’, ‘agent’ or ‘victim’ in a task where the commander coerced the agent to deliver painful shocks to the ‘victim’. We used ‘intentional binding’ as an implicit measure of sense of agency in both commanders and agents, in conditions of coercion and free-choice. We observed a reduced sense of agency when agents received coercive instructions, relative to when they freely chose which action to execute. We also found that sense of agency in the commanders was reduced when they coerced agents to administer the shock on their behalf, relative to when they acted by themselves. This last effect was associated with the commander’s self-reported level on a psychopathy scale. Thus, coercion resulted in neither commander nor agent feeling agency for the effect of the action, as measured through implicit methods. Our results could have profound implications for social decision-making and social regulation of moral behaviour.

## Introduction

All human societies require and endorse individual responsibility: people are aware of their actions and their consequences. While all, or almost all, human actions have some external consequences, actions made in a social context are particularly important because their effects impact not only the agent herself but also other people. Thus, the feeling of responsibility is a central aspect of the experience of agency in social contexts, and could be considered as a precursor to morality [1]. The *experience* of responsibility is a core aspect of the mechanism that constrains people to behave responsibly. At the same time, social factors can also constrain responsibility: sometimes, individuals are expected to obey coercive orders. For example, people under military command are expected to obey orders unconditionally, and their responsibility is not assessed in the usual way. The continuous balance between taking full responsibility for individual actions, and passing responsibility to commanding authorities, goes to the heart of how most societies work.

Nonetheless, history shows that the effects of coercion on the experience of responsibility can profoundly affect moral behaviour [2]. Stanley Milgram famously conducted a series of experiments that profoundly modified views of responsibility [3–4]. Milgram found that most people could be coerced into administering what appeared to be painful punishments. However, those experiments could not reveal *how* coercion influences behaviour. Today, the mechanisms of coercion remain poorly investigated.

We [5] recently measured the subjective experience of acting under coercion, and the effect of compliance on neural processing. In one condition, an experimenter ordered one participant to make a manual action that caused both an auditory tone, and either a simultaneous financial penalty or a calibrated painful electric shock to a co-participant. These harmful outcomes produced a financial gain for the participant. In another condition, participants chose for themselves how often to inflict the harm, and thus how much financial gain to obtain. Results showed that the social *context* of coercion reduced people’s individual sense of agency over their actions, as measured by the intentional binding effect, an implicit measure showing compression of perceived temporal intervals between an action and its outcome [6,7], relative to a free-choice condition. Explicit ratings of responsibility also showed a reduced feeling of responsibility under coercion. In addition, we found that event-related potentials to the tone exhibited reduced amplitude in the coercive condition, compared to a condition where participants freely chose which action to perform. These results confirmed the potential for social situations to modulate one’s own sense of agency, and thus moral conduct.

Acting under coercion therefore reduces the feelings of both agency and responsibility for one’s own actions. But are the sense of agency and responsibility then passed on to the person who gives the orders, as social and legal concepts of a chain of command might imply (i.e., “passing the buck”), or do they simply diffuse and disappear [8]? For example, military officers are held responsible for the actions of men they command, and government ministers are (in principle, if not always in practice) held responsible for the actions of civil servants working in their departments. In the present experiment, we assessed what happens to the feelings of agency and responsibility when one person commands another what to do.

To distinguish between these possibilities, we developed a paradigm in which three volunteer participants were tested together, each playing in turn the roles of commander, agent and “victim”. To assess the experience of responsibility and agency of each participant, we used both implicit and explicit ratings. Feeling of responsibility were evaluated through explicit ratings [1]. To assess the implicit sense of agency, we used an established behavioural proxy based on estimated action-outcome intervals (‘intentional binding effect’, [6,9]), rather than explicit self-reports–since the latter are strongly influenced by social desirability biases [10] and do involve different psychological processes [11]. In the intentional binding paradigm, participants have to estimate the delay between their action (i.e. a keypress) and an outcome (i.e. a tone). If the movement is voluntary, the perceived time is shorter than in a condition in which the movement is involuntary (for instance, triggered by Transcranial Magnetic Stimulation (TMS) pulse over the motor cortex, [6]). This suggests that identifying oneself as the author of an action modifies time perception, by reducing it (see [12] for a review). Thus, in our study, we focus on whether coercion increases the perceived interval between an action and its outcome, i.e., reduces the sense of agency.

According to a “socially optimistic” hypothesis, when commanders give coercive orders that are followed by the agents, the commanders should feel a strong sense of agency and responsibility, even if agents do not. Commanders should thus judge that action-outcome intervals are short relative to a condition where they simply observe the agent choosing and making the action and report high responsibility ratings. According to an alternative “socially pessimistic” hypothesis, no difference between these two situations is expected–when one person gets another to do something for her/him, neither person actually feels agency and responsibility for the outcome. In this scenario, the person who issues the orders would not feel responsible because even though she decides how to act, she does not actually carry out the act herself. The person who executes the orders would likewise not feel agency and responsibility for the outcomes because even though she carries out the action, she is not the one who chose which action was to be carried out.

Such issues—agency in social chains—, was explored in a previous study. Pfister et al. [13] investigated whether performing an action (= leader) that leads a second agent (= follower) to perform another action could influence implicit SoA. There was no evidence of subjective compression of time, neither for leaders nor for followers, over the final outcome of the chain (see their Experiment 2), which is consistent with our ‘socially pessimistic hypothesis’. However, in their paradigm, the leaders had no strong decisional power, since they could only press a single key when they wanted to do, a key that had no ecological consequences in the external world. This may have thus reduced their SoA over the final consequence of the chain. In contrast, the commander in our experiment could freely decide what order to give and when to give it [14], thus controlling the action of the follower, and hence potentially influencing sense of agency. Given that the research questions examined in the present study have been poorly investigated in previous literature, we also included some more exploratory analyses, in an effort to relate our sense of agency measures to other relevant psychological variables, such as personality traits as measured through self-report questionnaires.

## Experiment 1

### Experiment 1: Method

#### Materials and procedure

Participants completed several questionnaires online at least three days before participating: The Interpersonal Reactivity Index (IRI, [15]), the Levenson self-report psychopathy scale (LRSP, [16]) and the Social Dominance Orientation scale (SDO, [17]). The order of the questionnaires was counterbalanced across participants.

On arrival at the experimental laboratory, participants read an information sheet about the experimental procedure and the aim of the experiment. The three co-participants signed their individual consent forms simultaneously, ensuring that they were all aware of the others’ consent. Roles were assigned based on where participants chose to sit when they arrived in the room. One participant was the commander, another one was the agent and the third one was the “victim”. These roles were switched during the experiment, making the procedure fully reciprocal. The agent and the “victim” were sat at a table, face to face. A keyboard was placed between them, oriented towards the agent but visible by all participants. The commander sat at another, taller, table, placed near to the agent. The experimental task was controlled by a computer located on the agent’s right side, with the screen visible only to the agent and to the commander. The agent was instructed to press one out of two keys on the keyboard at a time she chose after the start of the trial, using either the right index finger or the right middle finger. This caused a tone to occur. The delay between key press and tone was set to vary randomly between 200, 500, and 800 ms. If a shock was delivered, the shock occurred at the same time as the tone. The commander and agent then both made independent estimates of the delay between the agent’s key press and the tone. The ‘victim’ also completed the task of interval estimates in order to keep them engaged during the task, and to reinforce the symmetry of the situation. However, our research questions focussed on the experience of agents and commanders, so the data from the ‘victim’ were not further analysed. They were informed that the delay would vary randomly on a trial-by-trial basis, between 0 and 1,000 ms (they were reminded that 1,000 ms makes 1 s). Participants were also told to make use of all possible numbers between 1 and 1,000, as appropriate, using the full scale as appropriate, and avoiding rounding. Each participant received a paper sheet with 60 empty boxes for their time estimates in each condition of the task. Participants’ answers were hidden from view of the other participants by a barrier, so as to avoid one participant influencing another.

**Fig 1:**
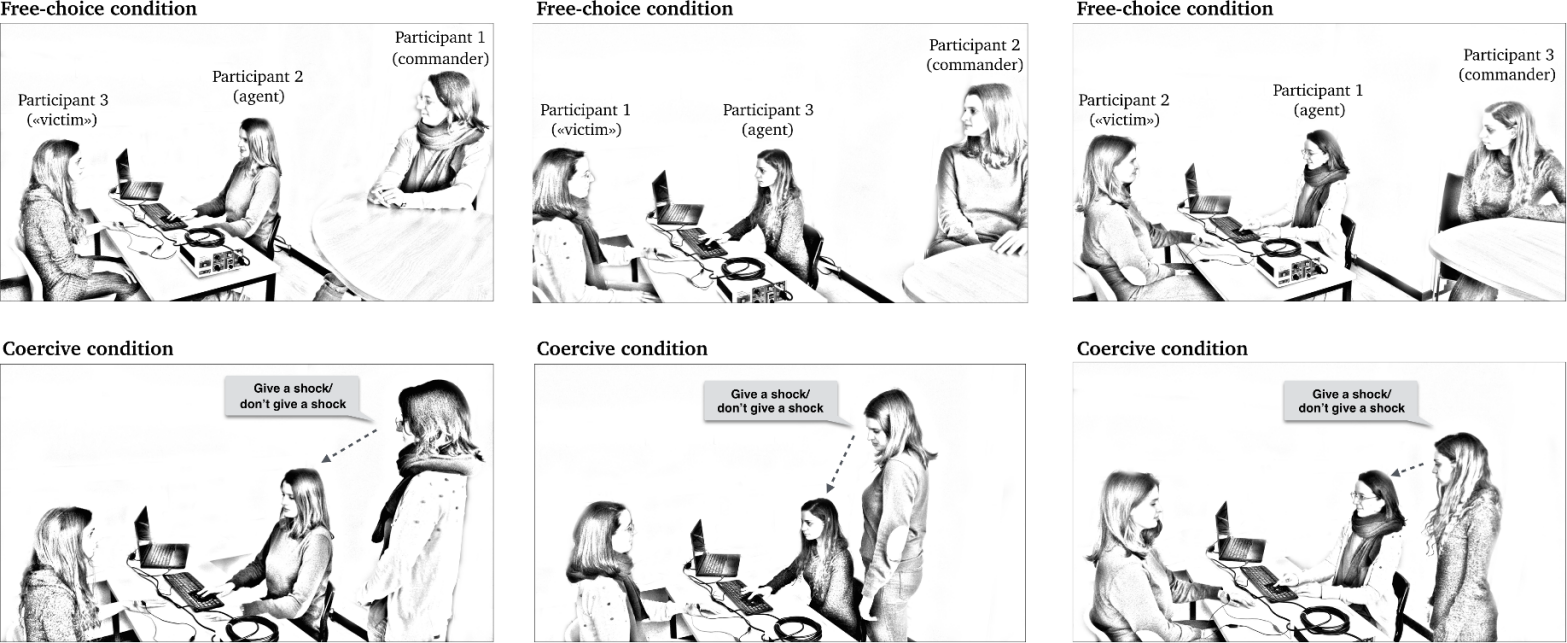
Experimental set-up of Experiment 1. In the free-choice condition, the agent was free to choose to deliver a shock or not (upper row). The commander sat at another table, passively watching. In the coercive condition, the commander was standing rather than seated, and ordered the agent to deliver a shock or not to the “victim” (lower row). Each participant took each role in turn (columns).

A short training session allowed participants to practice the interval estimation procedure. Two experimental conditions followed. All participants started with a specific endowment (€20). In the **free-choice condition**, agents were instructed that they could freely choose to increase their remuneration for the experiment by delivering a painful electric shock to the “victim”, using the appropriate key on the keyboard F to deliver a shock and H to not deliver the shock). Whether the agent delivered a shock or not, a similar tone occurred after the key press. They were told that they were totally free to choose how to act. The agents earned €0.05 each time they decided to deliver a painful electric shock to the “victim”. They earned no money if they decided not to deliver a shock. In this condition, the commander was simply a passive observer. In the **coercive condition**, the commander stood next to the agent and ordered the agent, on each trial, to deliver or not a painful electric shock to the “victim” (see **Fig 1**). The commander was completely free to decide which order to give, and could thus control the agent’s actions. If the commander ordered the agent to deliver a shock, both commander and agent earned €0.05. The experimenter sat in a corner of the experimental room, and never interfered with participants’ decisions.

There were 60 trials per condition (20 trials at each action-tone delay, in randomized order), giving a total of 360 trials (120 per roles). The order of the free-choice and coercive was counterbalanced across triads, but fixed within each triad.

Painful stimuli were delivered using a constant current stimulator (Digitimer DS7A) connected to two electrodes placed on the back of “victims”‘ left hand, visible to the agent and the commander. Individual pain thresholds were determined for all three participants after they had signed the consent form, and before starting the experiment. This threshold was determined by increasing stimulation in steps of 1 mA, following the procedure described in Caspar et al. [5] until participants reported a distinct pain sensation. The mean stimulation level selected by this procedure was 23.84 mA (SD = 7.14, pulse duration: 200 μs). All participants viewed the setting of each other’s thresholds.

In a post-session questionnaire, we asked participants to rate how responsible they felt during the experiment when they were the agent, and when they were the commander, as a percentage score. We also asked participants to describe other feelings during the experiment. The first item assessed how bad they had felt about giving shocks to the other participant, the second how sorry they were (answers were provided on 7-point Likert scales; “-3”; not very; “3”; very bad), the third how much they had looked at the other person’s face during the experiment (“-3”; not much; “3”; a lot) and how much pain they thought the other person had felt (“-3”; none at all; “3”; a lot). Participants had to answer both describing their experience as agent, and also as commander. In a first open question, participants were invited to explain if they administered more, less or a similar number of shocks to the “victim” when they were commander in comparison with when they were a free agent. They were invited to give open commentary about their decisions during the experiment. On a scale ranging from -3 (not very bad) to +3 (very bad), participants reported a mean score of .51 (SD = 1.73) when in the role of the agent and a mean score of .77 (SD = 1.81) when in the role of the commanders. This difference was not significant (*p* > .5). In a second open question, participants were invited to describe briefly their feelings and thoughts during the experiment. These descriptions confirmed that the participants were aware that their actions caused physical pain to their co-participant, and that they generally “felt bad” about doing so.

At the end of the experiment, participants were paid separately based on the original endowment, plus any additional financial gain during the experiment.

#### Participants

Thirty-six right-handed and naïve participants were recruited by triads. All participants were female to avoid possible effects of gender on relations of command and coercion. Our recruitment procedure included checks to ensure that participants were not close friends or relatives of others in their triad. To estimate the sample size, we used the effect size of Experiment 2 in Caspar et al. [5]. That study had an effect size *d*_z_ of 0.630 (based on the means and SDs of the within-subjects free-choice (mean: 367, SD: 119) and the coercive (mean: 426, SD: 131) conditions). To achieve a power of .90 for this effect size, the estimated sample size was 29 (GPower: [18]). Participants received between €20 and €29 for their participation. The following exclusion criteria were decided in advance of the experiment: failure to produce temporal estimates covarying monotonically with actual action-tone interval, or failure to follow instructions. To identify participants whom the action-tone intervals did not gradually increase with action-tone intervals, we performed linear trend analysis with contrast coefficients -1, 0, 1 for the three delays. One participant was excluded due to a non-significant linear trend analysis. The mean age was 21.57 (SD = 1.98). All participants provided written informed consent prior to the experiment. The study was approved by the local ethical committee of the Université libre de Bruxelles (008/2016), and was carried out in accordance with the approved guidelines. Individuals pictured on **Fig 1** were not real participants. They gave written informed consent (as outlined in PLOS consent form) to publish these case details.

### Experiment 1: Results

No participants withdrew from the experiment, and none reported any distress either after testing or at follow-up. During follow-ups, participants were invited to answer different questions about how they had lived this experiment in the different roles. Answers could be given by email or by phone (see Supporting Information S1 Text).

#### Number of shock*s*

**Fig 2:**
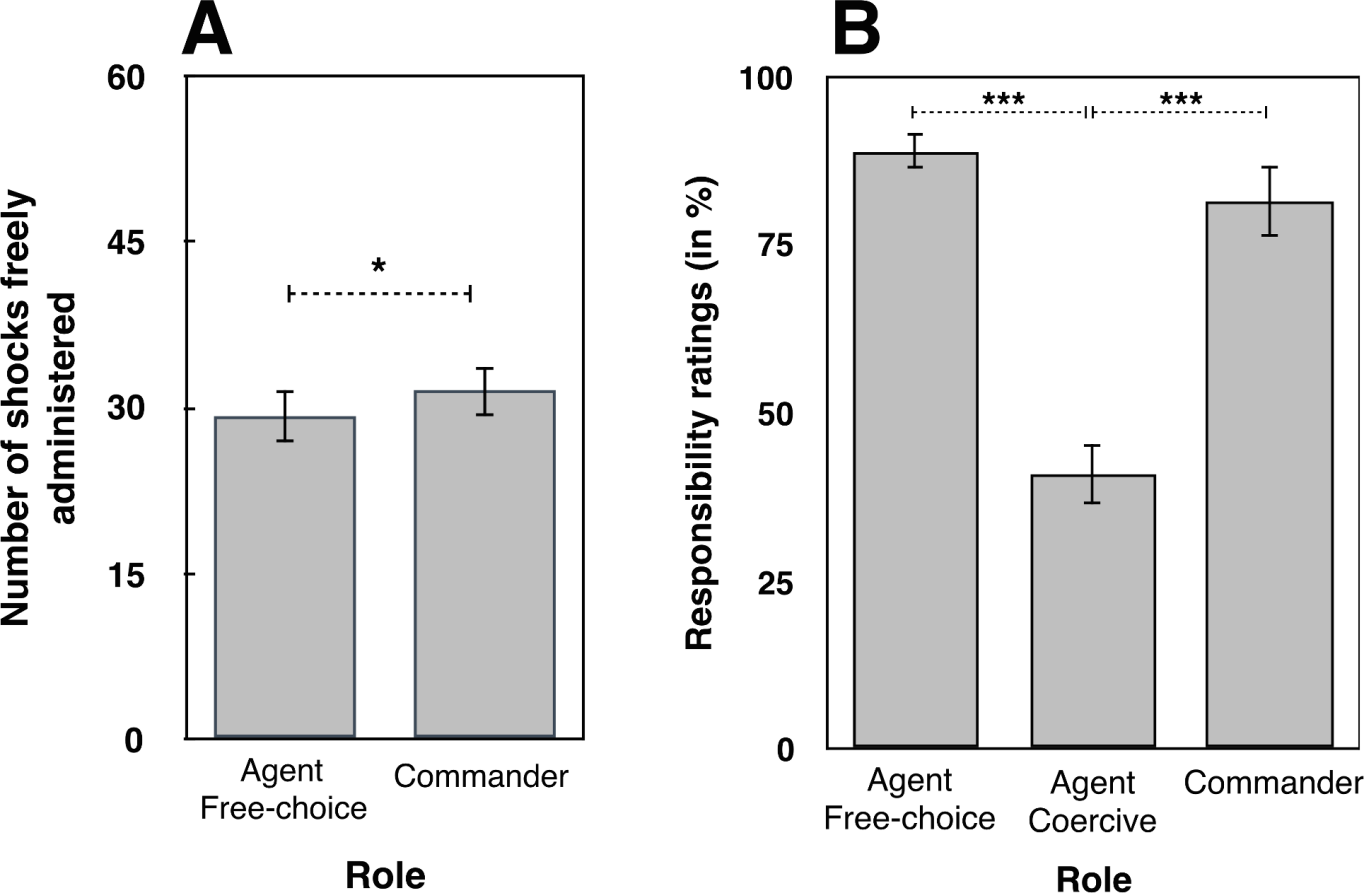
**A) Number of shocks.** Number of shocks freely administered by participants when they were in the roles of agent and of commander. **B) Responsibility ratings (%) in post-experiment questionnaires.** All tests were two-tailed. Errors bars represent standard errors. *** indicates a p value < = .001. * represents a p value between .01 and .05.

When participants acted as agents, they freely chose to administer painful electric shocks to the “victim” in 29.23/60 trials (CI_95_ = 24.58–33.87, min 1, max 60). When participants acted as commanders, they freely chose to administer painful electric shock to the “victim” in 31.57/60 trials (CI_95_ = 26.89–36.25, min 3, max 60). Because each participant undertook both roles, we compared these choices directly with a dependent-samples t-test. The difference was significant (t(34) = -2.090, *p* = .044, Cohen’s d = .353, see **Fig 2A**), thus showing that participants delivered more shocks to the ‘victim’ when they were commanders than when they were agents.

#### Responsibility ratings

Participants gave high responsibility ratings when they were free agents (Mean 89%, CI_95_ = 83–94) and also when they were free commanders (Mean 81.5%, CI_95_ = 72–91). The difference between these roles was not significant (*p* > .1). In contrast, participants reported lower responsibility when they received orders (mean 41%, CI_95_ = 30–52) compare to when they were free to decide which key to press (t(33) = 7.992, *p* < .001, Cohen’s d = 1.37) or free commander roles (t(33) = 5.296, *p* < .001, Cohen’s d = .908, see **Fig 2B**).

#### Interval estimates

**Fig 3:**
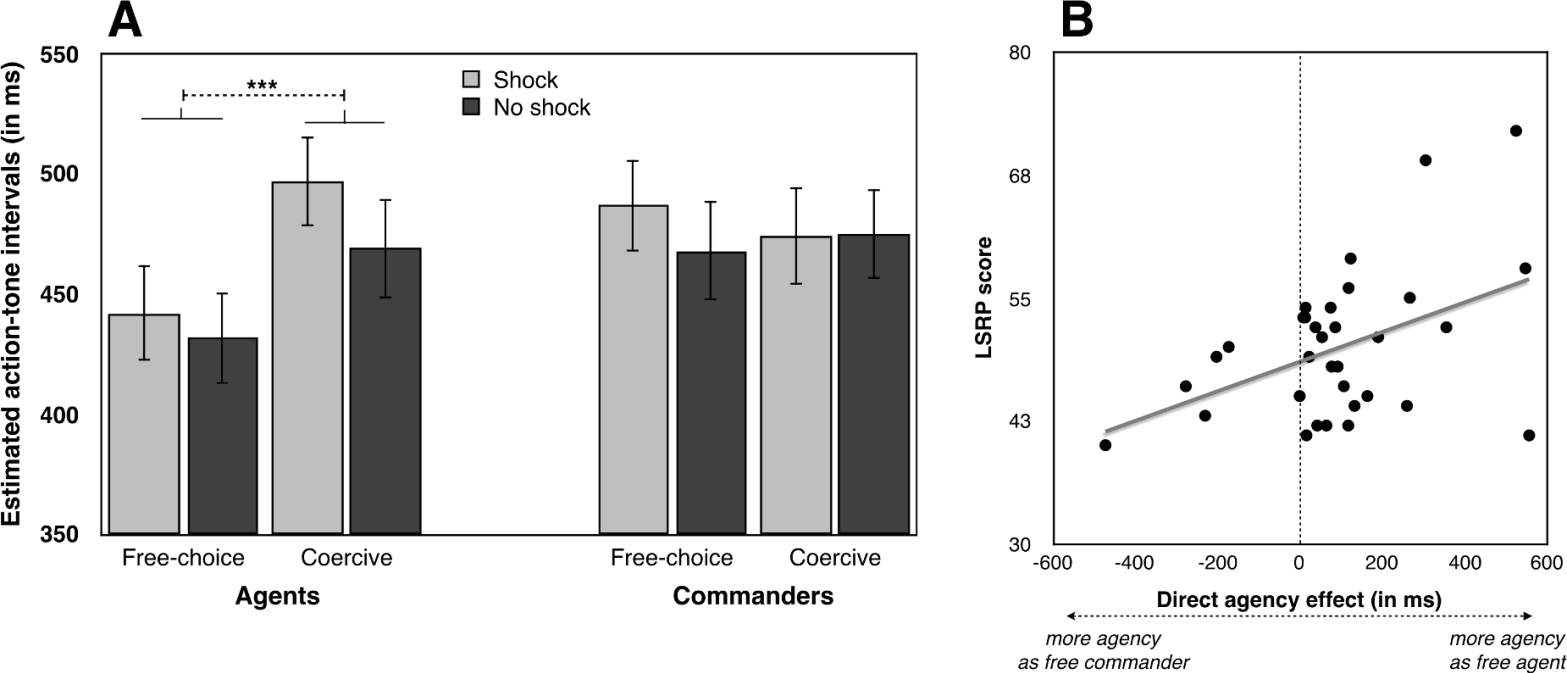
**A) Interval estimates.** Estimated action-tone intervals (ms) in free-choice and the coercive conditions for agents and commanders, pooling across the three action-outcome delays. The conditions have been named according to the agent’s perspective: in the free-choice condition, the agent was free to choose what to do and the commander was simply a passive observer. In the coercive condition, the agent followed the commander’s coercive instruction, while the commander was free to decide which order to give. **B). Correlation.** Correlation between LRSP score and the effect of indirect agency, defined as the difference between interval estimates of commanders vs agents in free choice conditions). All tests were two-tailed. Errors bars represent standard errors. *** indicates a p value < = .001. * represents a p value between .01 and .05.

We analysed both commanders’ and agents’ interval estimates using a Type III unbalanced repeated-measures ANOVA, with Role (Agent, Commander), Condition (Free-choice, Coercive) and Outcome (Shock, No shock) as within-subject factors and Order of the role as a between-subjects factor (see **Fig 3A**). This design was unbalanced, with 3 participants (8.5% of the total sample) had no data in at least one condition, because they chose either to deliver no shocks at all, or to deliver only shocks. The main effect of Role was not significant (*p* > .4), suggesting that interval estimates did not differ when participants were agents or commanders. The main effect of Condition was significant (F(1,29) = 8.254, *p* = .008, η^2^_***partial***_ = .222), with coercion leading to longer interval estimates (implying less sense of agency) than free choice (482 ms, CI_95_ = 442–522 and 457 ms, CI_95_ = 420–493), suggesting that coercion reduces the implicit feeling of agency, as measured through this method. The main effect of Outcome was also significant (F(1,29) = 4.723, *p* = .038, η^2^_***partial***_ = .140), with longer interval estimates when a painful outcome was given (476 ms, CI_95_ = 439–513) than when no painful outcome was delivered (463 ms, CI_95_ = 424–502). Most importantly for our hypothesis, the interaction Role x Condition was significant (F(1,29) = 7.640, *p* = .01, η^2^_***partial***_ = .209). Paired comparisons indicated that interval estimates were shorter in the free-choice (437 ms, CI_95_ = 397–477) than in the coercive condition (487 ms, CI_95_ = 445–528) for agents (t(34) = 4.343, *p* < .001, Cohen’s d = .734), but that this difference (free-choice: 475 ms, CI_95_ = 432–518 –coercive: 477 ms, CI_95_ = 433–520) was not significant for commanders (*p* > .3). To ensure that the absence of statistical difference was not due to lack of sensitivity in our data, we computed Bayes Factors (BFs, [19]) to refine these results. A BF between 1/3 and 3 indicates a lack of sensitivity. A BF below 1/3 or above 3 is typically interpreted as supporting for the null hypothesis, or for the alternative hypothesis, respectively. To compute a BF, we used Experiment 2 of Caspar et al. [5]. We selected this experiment because it is the first study published which compared a free-choice and a coercive condition in a paradigm involving electric shocks, and an implicit measure of sense of agency. No previous studies investigated SoA for commanders in a similar paradigm, so we assumed that this previous study of the agent role would provide a reliable basis to compute BFs for individuals from a similar population playing a commander role. To calculate the BF, we computed the expected mean and SD of the difference between the free-choice condition and the coercive condition [mean of plausibility(population value/theory) = 56 ms] and SD of plausibility(population value/theory) = 61 ms). The BF was 0.23, thus lending support to H0. This suggests that coercing another individual did not differ from passively observing the situation in terms of our sense of agency measure. The same interaction can also be considered by comparing the two conditions in which agents/commanders had full decisional power (the free-choice condition for agents and the coercive condition for commanders): in this case, interval estimates were shorter for agents than for commanders (t(34) = 2.331, *p* = .026, Cohen’s d = .394). When agents and commanders had no decisional power (i.e., the coercive condition for agents and the free-choice for commanders), their interval estimates did not differ (*p* > .4). The other interactions were not significant (all *p*s > .1). Neither the main effect of Order (*p* > .8), nor its interaction with other factors (all *p*s > .3) were significant. A series of additional analyses were then conducted. Given that participants were free to decide the number of harmful actions to deliver when they were agents and commanders, we also reanalysed the data using this information (number of shocks freely delivered when commanders–number of shock freely deliver when agents) as a covariate. This covariate was not significant (*p* > .9) and the general pattern of results was unchanged. We also reanalysed the data by correcting each participant’s interval estimates by subtracting the real action-tone delay from the judged action-tone delay for each trial in order to control the unbalanced distribution of delays (200, 500, 800 ms) across within-subject factors (see S1 Fig). The general pattern of results was unchanged (see Section a in S2 Text), but the Outcome factor was no longer significant (*p* > .5). Finally, we additionally conducted further analyses including Delay between the agent’s keypress and the tone as a within-subject factor in order to investigate the effects of duration of the action-outcome intervals. Section b in S2 Text shows the analyses, which mainly showed that the coercion effect for agents was stronger for long action-effect intervals than for short action-effect intervals.

We conducted further exploratory analyses. To assess whether or not some self-reported personality traits could influence the “coercion effect” (i.e., the difference between the free-choice and the coercive condition) a forced-entry regression method was used to fit a multiple linear regression model. We centred the overall score of all predictor variables (IRI, LRSP and SDO) before building the model. Two participants did not answer all the questions of the online questionnaire, so their data were not taken into account. We observed that none of the predictor variables modified the coercion effect, neither for agents (all *p*s > .1), nor for commanders (all *p*s > .1). S1 and S2 Tables show supplemental results for similar correlations performed with the individual subscales of these instruments, where available.

We also investigated the effect of personality on an estimate of the “direct agency effect”, defined as difference in interval estimates when freely chose as a commander (and coerced another person to enact their choice), relative to when they chose and acted directly as an agent. A negative score on this difference measure indicates that interval estimates were shorter, and thus sense of agency stronger, when commanding others, compared to acting directly. Given that multiple correlations were performed, we applied a Bonferroni correction (α/3 = 0.016). We found a strong relation between LSRP score and the direct agency effect (t(32) = 2.921, *p* = .007, Beta = .497). The higher participants scored on LSRP (i.e., more psychopathic traits), the less they tended to feel a sense of agency when making free choices in the commander role, relative to the direct agent role—producing a positive score on our indirect agency measure (see **Fig 3B**). Importantly, the same analyses conducted on the corrected interval estimates (real action-tone delay—judged action-tone delay for each trial) also lead to similar results, with a strong correlation only with the LSRP (t(32) = 3.106, *p* = .004, Beta = .531). Supplementary analyses revealed that the secondary psychopathy subscale (impulsivity, poor behavioural control) of the LSRP was a better predictor (t(32) = 3.263, *p* = .003, Beta = .491) than the primary psychopathy subscale (also named ‘callousness’, *p* > .3). Other predictors (IRI, SDO) were not significant (all *p*s > .05). S3 Table shows results for similar regressions for specific subscales. We additionally investigated whether or not our direct agency measure could predict the difference between the number of shocks administered by agents and commanders, but we found no significant relation (*p* > .8).

### Experiment 1: Discussion

Hierarchies frequently involves a form a diffusion of responsibility between those who make decisions, and those who translate them into action. To our knowledge, the present study is the first attempting to systematically explore how individuals’ sense of agency varies between the role of decider and the role of executor in an ecological paradigm.

First, we observed that agents reported longer interval estimates in the coercive condition than in the free-choice condition, replicating previous results [5]. Importantly, in this experiment, the orders were not given by an experimenter as previously, but rather by another participant who was free to decide which order to give. This suggests that the reduction of the implicit experience of agency is a common feature of coercive situations. The basic effects of coercion on sense of agency do not require a social structure or institution of “authority” [4]. Rather, coercive modulation of agency may arise whenever individuals find themselves in low-power relations [20], even when these are transient and reversing, as here. This finding adds generality and ecological validity to previous reports of coercion effects on sense of agency [5]. We additionally found a modest but significant main effect of outcome, with longer interval estimates, implying lower sense of agency, when actions produced shocks compared to when they did not. Reduced sense of agency for negative outcomes has been reported previously, using event timing estimates [21–22], although this effect was not statistically significant in our previous study with painful outcomes [5]. We speculate that the size, and thus significance, of this effect may depend on differences between experiments in the emotional valence associated with the outcomes (negative vocalisations versus painful electric shocks associated with a monetary reward in [22] and [5] respectively).

Second, we observed a striking increase in interval estimates, corresponding to a reduced sense of agency, when participants’ instructed another person to carry out their intentions, compared to when they themselves made the action. When commanders coerced another participant to implement their free choices, rather than doing it themselves, they appeared to distance themselves from the outcome of their intentions, and felt less agency. Our implicit measures showed lower sense of agency for such indirect, mediated coercive actions than for direct freely-chosen actions. Interestingly, exploratory analyses revealed that this effect was significantly related to individuals’ psychopathy scores. The higher participants scored on primary psychopathy, the greater the difference between interval estimates when coercing another agent, vs when acting as free agent oneself was. Thus, we may have identified an important trait variable relevant to the diffusion of responsibility that occurs when people coerce others, which should be confirmed in future studies.

Interestingly, we found a dissociation between implicit feeling of agency and explicit report of responsibility for commanders, but not for agents, in Experiment 1. Commanders reported high responsibility scores when they gave orders to the agent, but showed greater interval estimates, implying a reduced sense of agency, relative to performing an action themselves. This dissociation might be explained by the fact that explicit measures are strongly biased by social desirability factors: commanders know that they *should* feel responsibility and thus report that they do feel it, even though they may not actually feel it. Implicit measures of agency are less influenced by such demand factors. Importantly, the commanders’ high explicit responsibility ratings confirm that they understood the psychological issues associated with the role of commanding.

Our first experiment involved an important confound between low-level and high-level features of social roles. In particular, our participants made manual actions to trigger the tones and shocks when they acted as agents, but merely gave spoken instructions, which ultimately and indirectly lead to tones and shocks, when they acted as commanders. Thus, the difference between agent and commander roles is confounded with the direct causation of the outcome. The vast majority of sense of agency studies, particularly those using implicit measures, have focused on direct causal control over outcomes via manual actions [6,23–24]. Therefore, the lower sense of agency for commanders rather than agents might reflect an absence of manual action and direct causation, rather than a specific effect of social role. In situations of coercion, commanders indeed do not act by themselves. Rather, they impose their decisions onto a third party, who actually executes them. In neurocognitive terms, coercion splits the action control system across two individuals, so that the higher cognitive processes of action selection take place in the commander’s brain, while the lower-level motoric processes of action execution take place in the executor’s brain. Previous studies have highlighted the importance of sensorimotor signals for the sense of agency [25–29]. We suggest that commanders’ reduced sense of agency when they coerce others in Experiment 1 may reflect the lack of the normal sensorimotor information that underpins experiences of direct agency.

In a second experiment, we tested this hypothesis directly, by introducing a situation where the commander gave their coercive instructions not by verbal instruction as in Experiment 1, but by a manual action that more directly lead to the shock. In order to control for the presence of manual actions in all the experimental conditions, commanders had to press a key that delivered an instruction also in the free-choice condition. To preserve a feeling of freedom for agents, we thus told them that they could freely decide to follow or not this command. Indirectly, we thus offered the possibility to agents to obey or disobey the commander’s instructions. Previous research showed that an increased effort [30] and an increase in the number of alternative actions [31] both increase the feeling of agency. Choosing to go against coercive instructions should therefore lead to a greater sense of agency than being coerced. This modification also allowed us to control for causal events preceding the agent’s keypress [32]. Indeed, one could assume that the reduction of agency in the coercive condition for agents is due to the occurrence of some event (i.e. the commander’s instruction) preceding the keypress, rather than due to the coercive content of that event *per se*. This additional antecedent event is not present in free-choice conditions, thus potentially explaining the difference between conditions. However, experiment 2 balances this potential antecedent event across conditions. Specifically, in the free-choice condition of Experiment 2, commanders instructed the agents when to press a key (just as in the coercion condition), but the agents were free to decide whether to follow the instruction or not. Thus, in experiment 2, coercion and choice were different contexts, but the events were identical across conditions.

Finally, we also included a ‘predictability’ factor in the coercive condition in Experiment 2: the instruction that the commander sent by pressing one of two keys was either fully predictable from the key pressed (coercive predictable condition) or only predictable on 50% of trials (coercive unpredictable condition). Previous studies have showed that unpredicted outcomes reduce SoA [33] and should thus constitute a ceiling effect in the reduction of agency.

Again, the socially optimistic hypothesis predicts shorter interval estimates when the commander’s orders are applied by the agent in comparison with a situation in which the agent does not follow those orders. We further predicted that the commander’s more direct motoric involvement in causing the shock would increase their feeling of agency when they gave orders that are executed by the agent. Importantly, in Experiment 2 we could not directly compare agents and commanders’ interval estimates since participants in each role judged the delay between their *own* motor action and the resulting tone. Thus, the action judged was not physically identical for agents and for commanders, and the action-outcome intervals differed. Some of the analyses performed in Experiment 1, namely the direct agency effect, could thus not be investigated in Experiment 2. However, whenever possible, we performed statistical analyses comparing Experiments 1 and 2.

## Experiment 2

### Experiment 2: Method

#### Materials and procedure

**Fig 4:**
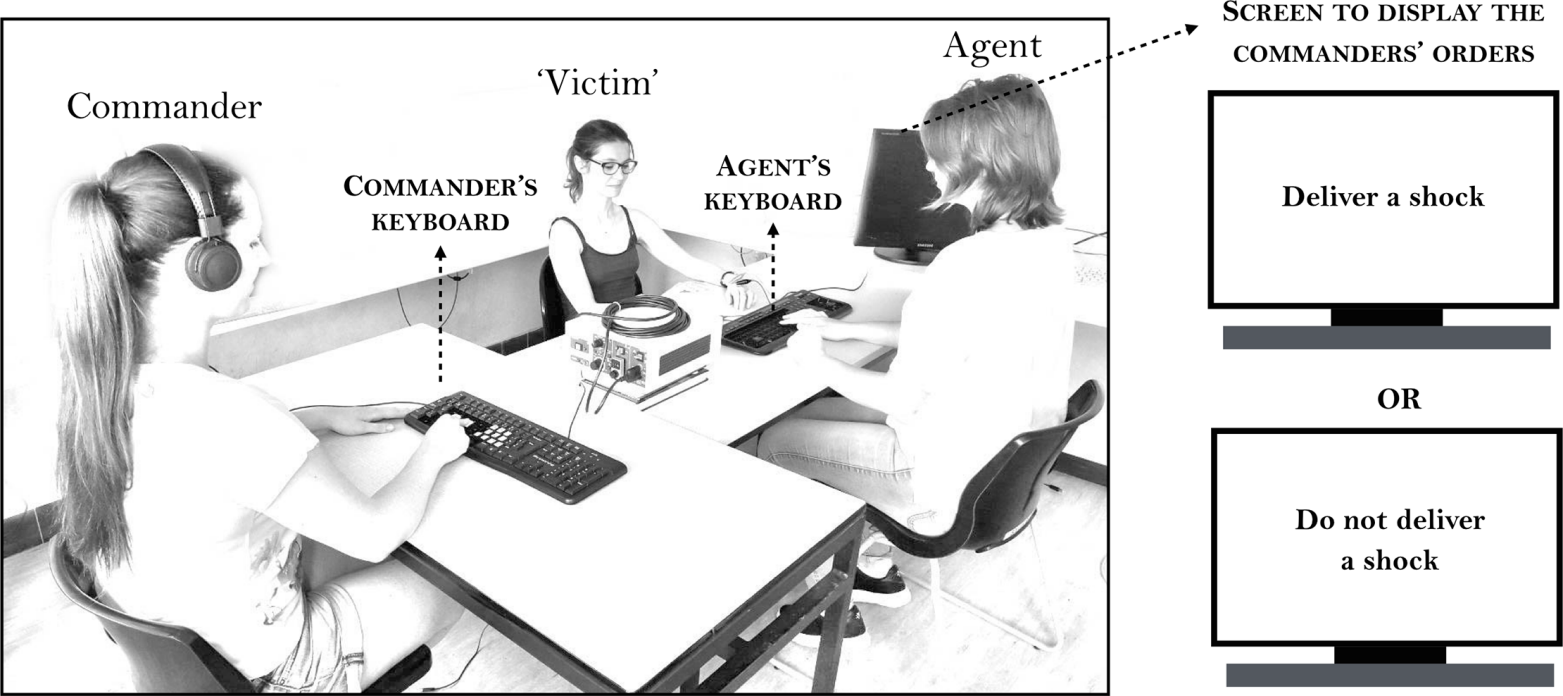
Experimental set-up of Experiment 2.

The procedure was globally similar to Experiment 1, with the same three reciprocal roles. In Experiment 2, the participant who was the commander sat at another table, similar in size to agent’s and “victim’s” table (see **Fig 4**). A keyboard was placed between the agent and the “victim”, oriented towards the agent but visible to all three participants. Another keyboard was placed in front of the commander, and was invisible to both the agent and the “victim”. The agent and commander could both see the screen running the task program. For both the commander and the agent, two keys on their respective keyboards were marked “shock” and “no shock”. The commander was instructed to press whichever of these two keys she wished, in her own time. This caused a coercive instruction to appear on the agent’s screen: “deliver a shock” or “do not deliver a shock”. The agent responded to this instruction by pressing the corresponding key (“shock” or “no shock”) on her own keyboard. After 200, 500 or 800 ms, a tone occurred. If a shock was delivered, the “victim” received the shock at the same time as the tone. The agents and “victims” both independently estimated the delay between the agent’s keypress and the tone. The commanders’ task was to estimate the delay between her own keypress and the tone. Participants were told that the delay would vary randomly but that the tone would always occur within 1 s of the agent’s action. No upper limit was mentioned for commanders’ interval estimates, because their delay depended on the delay for the agent to respond to the commander’s instruction displayed on the screen. Commanders were simply told that if they thought that the delay was exactly 1 second, then they could report 1000 ms on the answer sheet and if they thought that the delay was half a second, they could answer 500, etc. They were told that the interval could have any duration, and would vary from trial to trial. They were asked to avoid rounding their estimates. The commanders wore headphones during the entire experiment: this prevented them from hearing the agent’s keypress but nevertheless allowed them to hear the tone, as verified in a pilot test. In addition, we ensured that the agent’s keypress was not directly visible to the commander, by placing the electrical stimulator immediately in front of the agent’s response keys. We ensured that this was the case by asking commanders whether or not they could see the agent’s keypress. This procedure aimed to reduce perceptual bias for the commander’s interval estimates.

**Fig 5:**
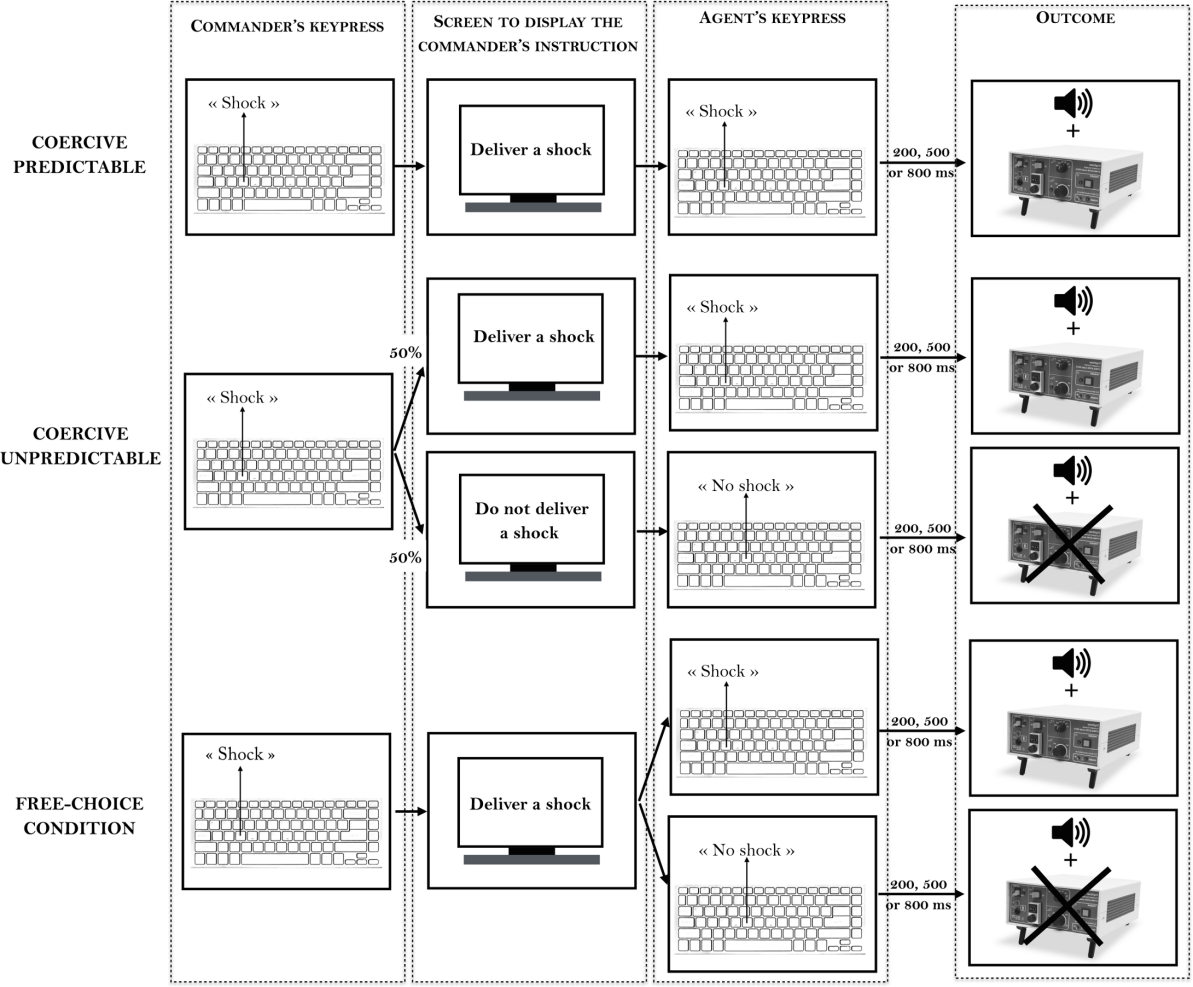
Schematic representation of the three experimental conditions, according to the role. In each case, the commander freely chose to administer a shock in the example trial shown.

The experiment began with a short training session, in which participants could practice the task and the interval estimate procedure, followed by three experimental conditions in counterbalanced order. In the **coercive predictable condition**, commanders were instructed that they were totally free to choose to deliver a shock to the “victim”, or not, by pressing the corresponding key on their keyboard. The commander’s decision was immediately displayed on the agent’s screen as a coercive instruction (for instance, if the commander pressed the key “shock”, the instruction “deliver a shock” was displayed on the screen, see **Fig 5**). The agent was instructed to always follow the instruction by pressing the corresponding key on their own keyboard. Both the commander and the agent earned €0.05 each time the commander decided to deliver a painful electric shock to the “victim”. In the **coercive unpredictable condition**, the commander could again freely choose which key to press, but the mapping between their choice and the instruction displayed to the agent was random. Participants were informed of this unpredictable mapping. The agents were instructed to follow the instructions displayed on the screen, and were aware that this might or might not match the commander’s decision on that trial or not. The commander could see if the instruction matched her keypress by viewing the screen on which financial reward for actual shock events was shown. Again, both the agent and the commander earned +€0.05 each time a shock was delivered to the “victim”, signalled by a visual feedback on the screen. Finally, In the **free-choice condition**, the commander again pressed one of the two keys to predictably display her order on the agent’s screen, but the agent was told that she could follow this instruction, or not, as she freely chose. In this condition, the €0.05 for delivering a shock was given only to the agent, not the commander. The experimenter sat in a corner of the experimental room, and did not interfere in any way with participants’ decisions.

There were 60 trials per condition (20 trials at each action-tone delay, in randomized order), giving a total of 540 trials (180 per role). The order of the three experimental conditions was counterbalanced during the experiment but the order of these conditions was the same within each triad.

The pain procedure was similar to Experiment 1. In Experiment 2, the mean stimulation level selected by this procedure was 31.53 mA (SD = 16.74, pulse duration: 200 μs).

In a post-session questionnaire, we asked participants to rate how responsible they felt during the experiment in each experimental conditions when they were agent and commander. The same questions as in Experiment 1 were asked. They were also invited to freely write a few words about how they felt and what they thought during the experiment. At the end of the experiment, participants were paid separately based on their earned financial gain during the experiment.

#### Participants

Thirty-three right-handed, naïve female participants were recruited by triads, as before. Participants received between €25 and €38.5 for their participation. Two participants were excluded due to a non-significant linear trend in analyses relating their interval estimates to the actual action-outcome interval. The mean age was 22.29 (SD = 2.55). All participants provided written informed consent prior to the experiment. The study was approved by the local ethical committee of the Université libre de Bruxelles (008/2016), and was carried out in accordance with the approved guidelines. Individuals pictured on Fig 3 were not real participants. They gave written informed consent (as outlined in PLOS consent form) to publish these case details.

### Experiment 2: Results

#### Number of shocks

**Fig 6:**
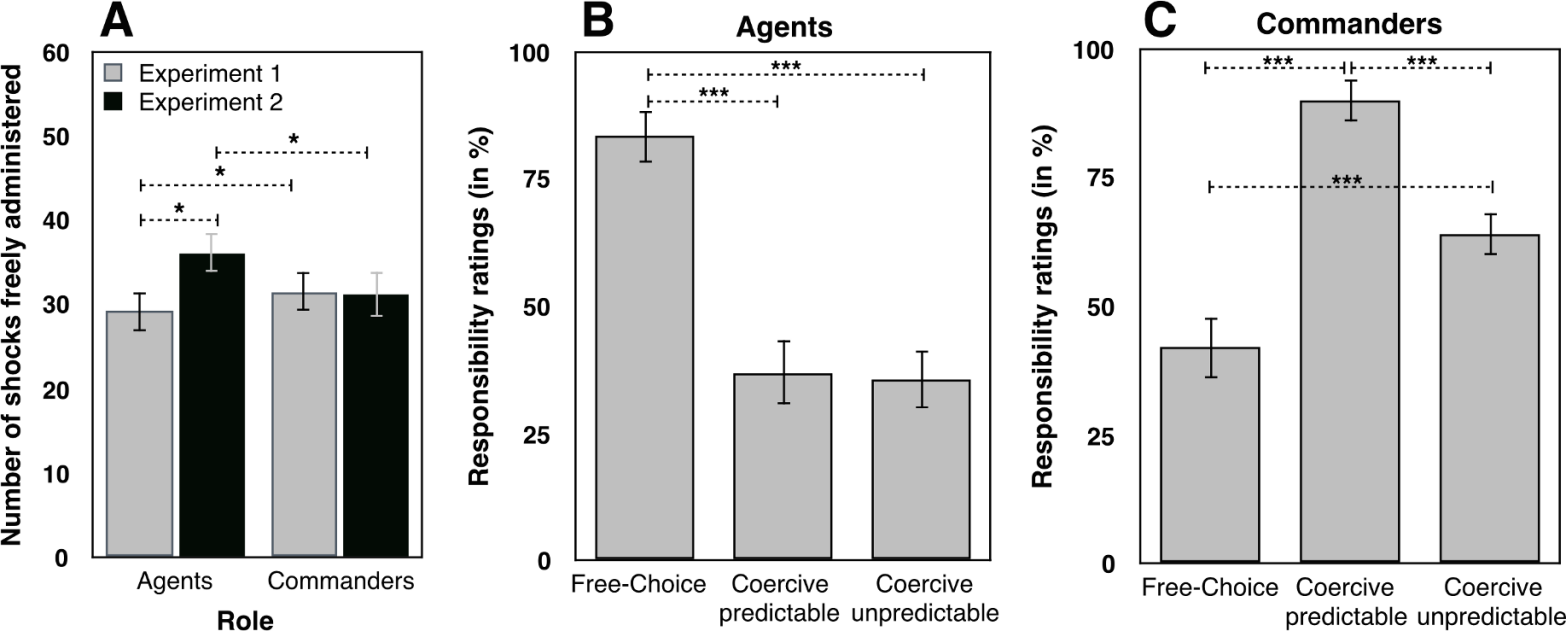
**A) Number of shocks.** Comparison of the number of shocks freely administered by agents (in the free-choice condition) and by commanders (in the coercive (predictable) condition) across Experiments 1 and 2. **B) Responsibility ratings of agents. C) Responsibility ratings of commanders.** All tests were two-tailed. Errors bars represent standard errors. *** indicates a p value < = .001. * represents a p value between .01 and .05.

When participants were agents, and thus made for themselves the manual action that could lead to the shock, they freely chose to administer painful electric shocks to the “victim” in 36.26/60 trials CI_95_ = 31.67–40.85, min 3, max 60). When participants were commanders and thus used the agent to apply their orders, they freely chose to administer painful electric shock to the “victim” in 31.32/60 trials (CI_95_ = 25.94–36.71, min 2, max 60). This difference was significant (t(30) = 2.592, *p* = .015, Cohen’s d = .465). Interestingly, the direction of the effect reversed the pattern seen in Experiment 1. We thus compared the number of shocks freely administered by agents and commanders in Experiments 1 and 2 (see **Fig 6A** for the differences between each experiment). A repeated-measures ANOVA with Role (agent, commander) as within-subject factor and Experiment (1,2) as between-subject factor was conducted on the number of shocks freely administered. Neither the main effect of role (*p* > .2), nor the main effect of experiment (*p* > .2) were significant. The interaction role x experiment was significant (F(1,64) = 11.450, *p* = .001, η^2^_***partial***_ = .152). Independent sample t-tests revealed that the number of shocks freely delivered by commanders did not differ between Experiment 1 and 2 (*p* > .9), suggesting that giving orders verbally or by the means of a keyboard did not influence the results. Rather, the difference between the two experiments lay in the free choices when in the agent role: Participants in Experiment 2 administered more shocks to the ‘victim’ in the free-choice condition than in Experiment 1 (t(64) = -2.183, *p* = .033, Cohen’s d = .762). Possible interpretations of this difference between experiments are given in the general discussion.

#### Responsibility ratings

We conducted a repeated-measures ANOVA on responsibility ratings with Role (Agent, Commander) and Condition (Free-choice, Coercive predictable, Coercive Unpredictable) as within-subject factors. The main effect of Role was significant (F(1,30) = 5.338, *p* = .028, η^2^_***partial***_ = .151), with higher responsibility ratings when participants were in the role of the commander (65%, CI_95_ = 58–72) then when they were in the role of the agent (52%, CI_95_ = 44–60). We also observed a main effect of Condition (F(2,60) = 52.402, *p* < .001, η^2^_***partial***_ = .636). The interaction Condition x Role was significant (F(2,60) = 5.148, *p* < .01, η^2^_***partial***_ = .146). When they were agents, participants reported feeling more responsible in the free-choice condition (83%, CI_95_ = 73–93) than in the coercive predictable condition (37%, CI_95_ = 24–49, t(30) = 6.289, *p* < .001, Cohen’s d = 1.12) and, than in the coercive unpredictable condition (36%, CI_95_ = 24–48, t(30) = 7.269, *p* < .001, Cohen’s d = 1.30). Responsibility ratings in the coercive predictable condition and the coercive unpredictable condition did not differ (*p* > .8), see **Fig 6B**. When they were commanders, participants reported feeling more responsible in the coercive predictable condition (90%, CI_95_ = 82–98, *p* > .2) than in the coercive unpredictable condition (64%, CI_95_ = 55–72, t(30) = 5.897, *p* < .001, Cohen’s d = 1.05) and, than in the free-choice condition (42%, CI_95_ = 30–54, t(30) = 7.054, *p* < .001, Cohen’s d = 1.26). Responsibility ratings were also higher in the coercive unpredictable condition than in the free-choice condition (t(30) = -3.981, *p* < .001, Cohen’s d = -.714), see **Fig 6C**. As in Experiment 1, responsibility ratings were similar when participants were free agents or free commanders (*p* > .2), thus showing that acting by one’s self or coercing another individual to carry out the action leads to a similar perceived responsibility.

#### Interval estimates

In Experiment 2, we could not directly compare agents and commanders’ interval estimates, since the intervals judged were different. To recap, agents judged the delay between their own keypress and the tone, while commanders had to judge the delay between their own keypress and the tone. Importantly, only the agent’s keypress led directly to the outcome, while the commander’s keypress did not. The commander’s keypress simply displayed the order on the agent’s screen. We therefore performed separate repeated-measures ANOVAs for the agents, and for the commanders.

**Fig 7:**
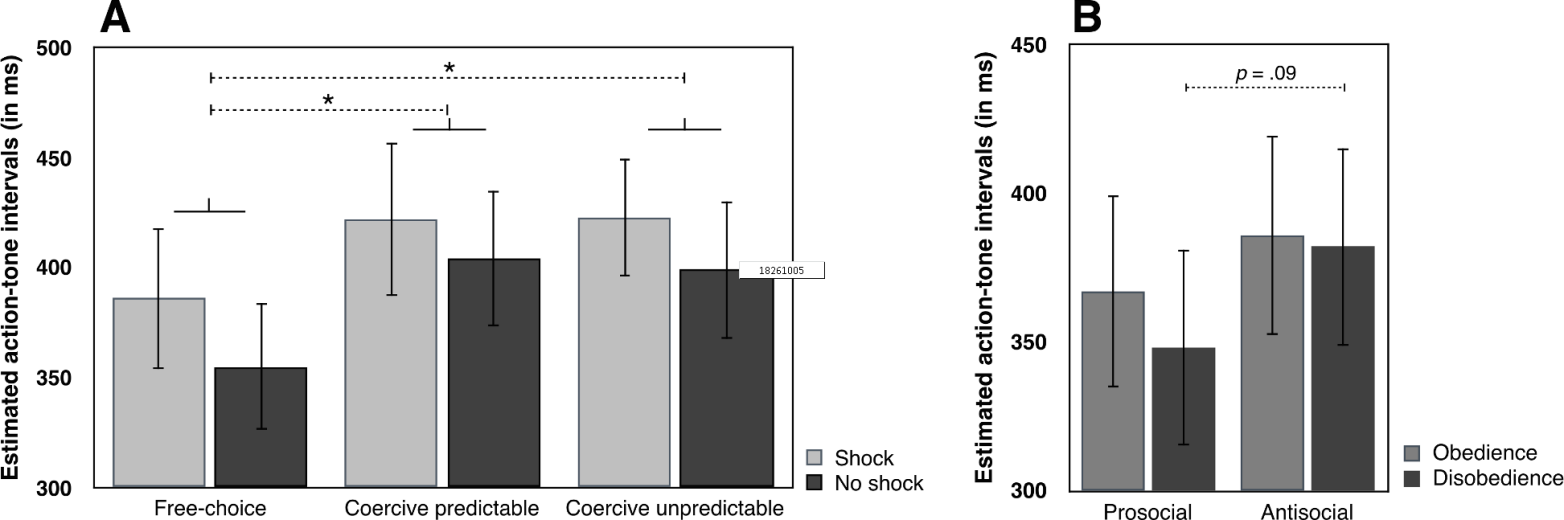
**A) Interval estimates for agents. B) Interval estimates for agents in the free-choice condition according to commanders’ instructions.** All tests were two-tailed. Errors bars represent standard errors. * represents a p value between .01 and .05.

We analysed agents’ interval estimates using a Type III unbalanced repeated-measures ANOVA, with Condition (Free-choice, Coercive Predictable, Coercive Unpredictable) and Outcome (Shock, No shock) as within-subject factors and Order of the role as a between-subjects factor (see **Fig 7A**). This design was unbalanced because 3 participants (9.6% of the total sample) had no data with or without shocks in at least one condition. The main effect of Condition was significant (F(2,52) = 3.643, *p* = .033, η^2^_***partial***_ = .123). Agents’ interval estimates were shorter, suggesting a stronger sense of agency, in the free-choice condition (372 ms, CI_95_ = 312–432) than in the coercive predictable condition (413 ms, CI_95_ = 350–476, t(28) = -2.482, *p* = .019, Cohen’s d = -.460) and, than the coercive unpredictable condition (411 ms, 95%CI = 353–470, t(29) = -2.311, *p* = .028, Cohen’s d = -.421). The coercive predictable condition and the coercive unpredictable condition did not differ (*p* > .9). The main effect of Outcome was significant (F(1,26) = 8.884, *p* = .006, η^2^_***partial***_ = .255), with longer interval estimates when a painful outcome was given (413 ms, CI_95_ = 354–472) than when no painful outcome was delivered (384.5 ms, CI_95_ = 328–440). The interaction Condition x Outcome was not significant (*p* > .7), nor the interactions with Order (all *p*s > .5). We repeated this analysis using the number of trials in which agents decided to disobey as a covariate, and found that the general pattern of results was unchanged. A forced-entry regression method was used to fit a multiple linear regression model. As in Experiment 1, we observed that none of the overall scores on the questionnaires predicted the coercion effect (defined as the difference between estimates in the coercive predictable condition–free-choice condition; all *p*s > .4). S4 Table describes the result of multiple linear regressions with each subscale.

We additionally investigated whether obeying vs disobeying the commander influenced agents’ interval estimates. We therefore conducted an unbalanced repeated-measures ANOVA with Obedience (Obedience, Disobedience) and Outcome (Shock, No shock). Because the frequency of disobedience varied widely (S2 Fig and S3 Text), percentage of disobedient trials was used as a covariate. Ten participants (32.2% of the total sample) had no data in at least one condition, due to the pattern of the commander’s instructions and/or their own choices to obey or disobey them. Neither the main effect of Obedience (*p* > .7), nor the main effect of Outcome (*p* > .9) were significant, but the interaction between them was (F(1,19) = 4.663, *p* = .044, η^2^_***partial***_ = .197, see **Fig 7B**). Interval estimates were shorter when participants refused to administer a shock when the commander requested to do so (329 ms, CI_95_ = 264–395), compared to when participants administered a shock while the commander requested not to do so (360 ms, CI_95_ = 291–429, t(21) = 1.776, *p* = .090). This result implies a modest trend towards increased sense of agency in conditions of prosocial vs antisocial disobedience. Conversely, when participants obeyed the commander’s instruction, there was no difference in interval estimates between trials with or without a shock (*p* > .2).

**Fig 8:**
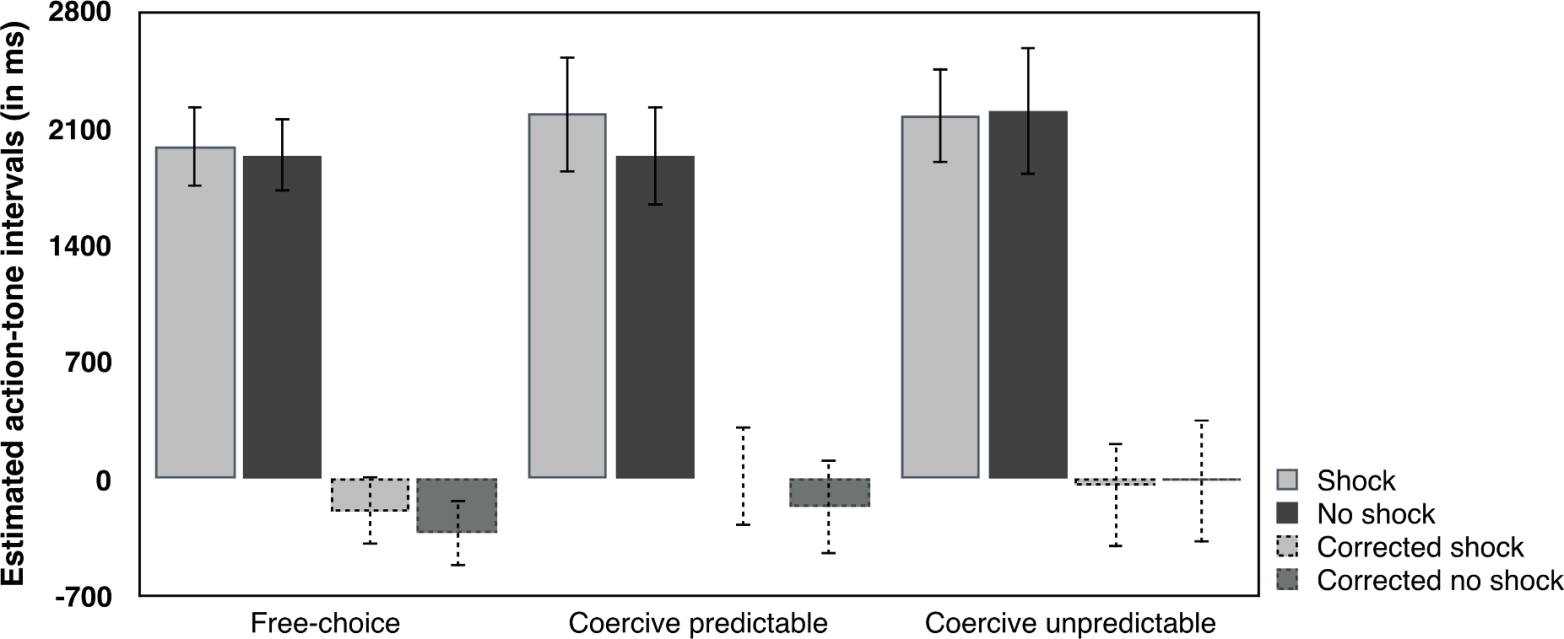
Interval estimates for commanders. All tests were two-tailed. Errors bars represent standard errors. * represents a p value between .01 and .05.

In Experiment 2, the actual action-tone intervals of commanders were not fully under our control since these intervals depended on when the agents pressed the key in response to the visual instruction sent by the commanders’ keypress. We therefore corrected the commanders’ interval estimates by subtracting the real action-tone delay from the judged action-tone delay for each trial. This produced a judgement error that could be compared between conditions. Note that an underestimate of the actual interval corresponds to a negative value. We then analysed commanders’ corrected interval estimates using a repeated-measures ANOVA, with Condition (Free-choice, Coercive Predictable, Coercive Unpredictable) and Outcome (Shock, No shock) as within-subject factors and Order as a between-subjects factor (see **Fig 8**). Neither the main effect of Condition (*p* > .3), nor the main effect of Outcome (*p* > .8), nor their interaction (*p* > .6) were significant. The interaction Outcome x Order was just significant (F(2,26) = 3.407, *p* = .049, η^2^_***partial***_ = .208) but not investigated further because it was not predicted from our experimental hypotheses. As in Experiment 1, none of the general score of self-reported personality questionnaires (LSRP, IRI, SDO) explained the coercion effect (all *p*s > .7). S6 Table in Supporting Information describes the results of multiple linear regression with each subscale.

### Experiment 2: Discussion

Behavioural results indicated that agents’ interval estimates were shorter in the free-choice condition than in the coercive predictable and the coercive unpredictable conditions. These results, in line with Experiment 1 and our previous study [5], again suggest that receiving orders reduces the implicit feeling of agency. It also suggests that the coercion effect cannot simply be an artefact of the presence of an additional instruction event in our first experiment [5]. Again, we found no interaction with the outcome factor. That is, free-choice conditions produced stronger intentional binding than coercion conditions, whether the action lead to a shock or not. In Experiment 1, we could not identify whether the reduced sense of agency displayed by commanders giving coercive orders, compared to agents making free choices was due to the lack of any manual sensorimotor signal when commanding, or due to the cognitive process of mediating one’s agency by coercing another individual. In Experiment 2, commanders gave their orders through a keyboard and had to judge the delay between their own keypress and the tone, thus controlling for the role of sensorimotor signals. Results showed that commanders’ interval estimates did not differ between the free-choice and the coercive conditions, being predictable or not thus supporting the socially pessimistic hypothesis. This suggests that the crucial difference between being an agent vs being a commander does not lie in the form of motor action (verbal or manual), but in the (social) role in determining the outcome for the ‘victim’. Importantly, the intervals judged by the commanders were longer and more variable than that those judged by agents, because the interval judged by the commanders included the agent’s reaction time to the instruction. In principle, therefore, we cannot exclude the possibility that commanders failed to show intentional binding simply because the time delays that they judged exceeded those at which intentional binding is normally found. However, this seems unlikely, since several studies have reported binding effects at delays comparable to those judged by the commanders, and indeed at delays up to 4 s [34]. It remains unclear whether intentional binding increases or decreases as delays increase, since both results have been reported [6,35]. Interval estimation and events timing methods may give different results in this regard. In our own data, using interval estimation, we found that the coercion effect for agents’ intentional binding was stronger at longer delays. This makes it unlikely that the absence of any significant coercion effect in the commanders simply reflected the longer intervals that they judged. Another reason for this reduction of agency for commanders may be that all participants knew in advance that they would play the three roles, namely commander, agent and ‘victim’. This role reversal procedure may have contributed to increase the perception of diffusion of responsibility and thus to a reduction of agency for each role. This could be tested in future between-subject experiments in which each participant plays only either the commander or the agent role.

In the free-choice condition of Experiment 2, agents could disobey the commander’s instructions. The results showed that choosing to resist coercive commands did not reliably boost the implicit sense of agency, even though this could represent a higher prosocial goal. Our agents were instructed that they could disobey whenever they wanted in the free-choice condition. In this sense, resisting the commander’s orders might be seen less as an act disobedience towards the commander, and more as an application of the instructions given by the experimenter. In addition, the disobedience rate was quite low overall (32%), and highly variable between participants. Thus, our design may have only limited ecological validity for studying disobedience in everyday settings. Future studies could develop new designs specifically to investigate resistance to coercion.

## General discussion

Previous studies showed that coercion reduces the primary experience of agency for agents [5,36]. The main objective of the present study was to investigate if SoA might be transferred from the coerced to the coercer in social situations. This hypothesis would imply that commanders themselves experience a strong implicit SoA and responsibility over the consequences of their orders, even if someone else actually performs the action. This hypothesis is ‘socially optimistic’ in the sense that *someone* at least retains a feeling of agency and responsibility in situations of coercion. It may have some echoes in social conventions such as the military tradition that the officer becomes responsible for the conduct of her or his soldiers, or the notion that parents are responsible for the behaviour of their young children.

In both Experiments 1 and 2, agents’ interval estimates were shorter in the free-choice condition than the coercive condition, thus confirming that obeying coercive commands reduces SoA even when the causal chain is controlled, thus replicating previous results. It additionally shows that the reduction of the implicit experience of agency under coercion does not depend on the social status of the person who gives the orders, but rather reflects a common feature of coercive situations.

Making a free choice between actions with harmful vs non-harmful impacts on another person thus increases the feeling of agency, compared to being instructed which action to choose. However, this boost in sense of agency with free choice was only present when participants directly executed the action through their own motor system, and were thus not only free, but also true agents. We indeed observed in Experiment 1 than when participants freely commanded someone else to make the action for them, the sense of agency was not boosted over and above a condition where they merely watched the agent choose. Experiment 1 only shows that the commander does not perceive SoA with respect to the agent’s action, since commanders had to judge the temporal delay between the agent’s keypress and the tone. Therefore, Experiment 1 rules out the simplest version of the socially optimistic hypothesis: we found that sense of agency is not simply transferred from the coerced to the coercer in situations of coercion. However, Experiment 1 does not reveal how commanders experience SoA over their *own* actions, but rather focused on commanders’ experience of the action of a coerced agent.

Therefore, in Experiment 2, we focused on the commanders’ experience of their own action. We observed that, when commanders estimate the delay between their own action and a tone, they do not experience a stronger SoA when they give coercive orders in comparison with a condition in which they give orders which are not truly coercive, in the sense that the agent may disobey them. In normal situations, choosing and executing an action gives lower interval estimates, implying stronger SoA, than merely executing it [37]. The situation of commanders seems to be an exception to this rule. Put another way, commanders’ sense of agency is not boosted when they choose between outcomes compared to when they do not, presumably because they do not actually execute the action themselves, but rely on the agent to do it for them. Importantly, this cannot be explained by a lack of comprehension of the experimental conditions, since the explicit ratings of responsibility of commanders showed that their explicit experience of responsibility decreased when they knew their commands could be disobeyed. Thus, Experiments 1 and 2 together suggest that having the power to decide which action to perform is not sufficient to produce a full sense of agency, and that ability to manually control the outcome (shock vs. no shock) is necessary for a full sense of agency to emerge. In other words, choosing an outcome, but delegating the motor action that directly causes it to another person does not produce a full sense of agency. This suggests that the psychological process of using another individual to carry out one’s own intentions reduces the experience of agency compared to directly making the action oneself, even when the initial motor action is comparable in both cases.

Interestingly, our exploratory analysis found that individual differences appear to modulate the reduction of agency when coercing someone else. Some individuals experienced similar feelings of agency when they were commanders and when they were free agents. Those individuals scored low at the Levenson self-reported psychopathy scale. Conversely, psychopathic traits appeared to increase the risk that people fail to feel responsible when in the commander role. The reported prevalence of high trait psychopathy in business leaders [38–39] may therefore seem worrying. However, given the exploratory nature of these analyses, future studies should pursue this question with a more targeted experimental design, and larger samples.

Many social species are organized according to a dominance hierarchy that greatly influences the persistence of the group [40]. While many nonhuman species are organized according to a linear hierarchy, humans frequently participate in multiple hierarchies, in which they play varying roles [41]. For instance, the same person may be both a mere employee in a professional context, but a dominant figure within a family or leisure circle. The hierarchical nature of human social structures has profound effects on responsibility for action, and on morality. Leaders frequently, and perhaps necessarily, delegate the execution of their decisions to others. Several types of evidence from moral psychology suggest this indirectness softens the subjective evaluation of harms committed [42–44]. In one variant of his seminal experiment, Milgram [4] observed that when participants did not administer the punishment themselves, but rather observed another person physically implementing their decision, obedience increased from 65% to 92.5%. One obvious mechanism may be that leaders who delegate execution to others may lack direct evidence about potential negative impacts of their decisions on others. For example, in political and social situations, leaders can get others to ‘do their dirty work’. However, our Experiment 1 found reduced sense of agency in leaders for both harmful and non-harmful outcomes, thus suggesting a more general effect.

Consistent with these theories, we observed in Experiment 1 that participants administered more painful electric shocks to the ‘victims’ when they played the role of commander than when they played the role of agent. This result confirms that indirect agency increases antisocial behaviours relative to direct agency, and that this tendency can be captured in laboratory settings. Interestingly, however, a simple change to the information protocol in Experiment 2 produced the reverse result: now participants inflicted more electric shocks to the ‘victims’ when they played the role of agents, than when the played the role of commanders. While this difference was not initially predicted, we believe it can be explained because of the difference in the information available to the victim in the two experiments. Experiment 1 was ‘informationally transparent’, in that all three participants knew, in every trial and condition, who was responsible for the decision to shock or not. In contrast, the actions in experiment 2 were untraceable by ‘victims’. That is, the ‘victims’ in Experiment 2 did not know whether the decision to harm or not was taken by the agent or by the commander, because they did not see the screen by which the commander communicated to the agent. Even in the ‘free-choice’ condition of Experiment 2, where the agent could disobey the commander’s instruction, the ‘victim’ had no way of knowing, on any given trial, whether they did so or not. The setup of the experiment ensured that the agent and the commander were both aware that the ‘victim’ could not know the source of the decision. We speculate that this lead agents to ‘hide’ their own decision behind the commanders’ instruction, unknown to the ‘victim’. For example, in the free-choice condition, the commander might transmit to the agent an instruction not to shock, but the agent might choose to disobey this instruction and actually shock the ‘victim’. In this case, the ‘victim’ could not know whether the shock they received had been decided by the commander or the agent. This lack of information may have allowed agents to diffuse their sense responsibility to the commanders–since the ‘victim’ could not know whether the agent was ‘only obeying orders’ of was acting voluntarily. This form of diffusion of responsibility might be described as ‘informational’: people may feel less responsibility when they know that the social context makes it difficult to recover whether their action was freely-chosen or not. Many famous historical and legal decisions turn on this problem.

In coercion situations, the cognitive functions of action choice and action execution are split across two separate individuals’ brains. One person chooses and commands, and another person acts accordingly. This represents a fundamental transformation of the classical model of human voluntary action, in which a person first decides, and then executes their actions. In the coercive situation, neither the agent nor the commander appear to experience agency for the outcome, as measured by our implicit ‘intentional binding’. Taken together, our results could be read as socially pessimistic for human voluntary action: coercive situations potentially undermine responsibility in both agents and commanders. However, our results also suggest high contextual sensitivity of sense of agency since exploratory analyses showed that self-reported personality traits modulate SoA of commanders when giving orders. This raises the interesting possibility of training the sense of responsibility–embedding such training into society, for example through education, could be protective against the potential moral hazards of loss of responsibility through coercion.

## Supporting information

S1 Text Debriefing mail. (DOCX)

S2 Text Section a) EXPERIMENT 1. Repeated-measures ANOVA with Role (Agent, Commander), Condition (Free-choice, Coercive), Outcome (Shock, No shock) as within-subject factors and Order of the role as a between-subjects factor on corrected interval estimates. Section b) EXPERIMENT 1. Repeated-measures ANOVA with Role (Agent, Commander), Condition (Free-choice, Coercive), Outcome (Shock, No shock) and Delay (200, 500, 800 ms) as within-subject factors and Order of the role as a between-subjects factor on corrected interval estimates.
(DOCX)

S3 Text EXPERIMENT 2. Disobedience rates.
(DOCX)

S1 Table EXPERIMENT 1. Multiple linear regression coefficients with each subscale of the questionnaires as the independent variables and the “coercion effect” of agents as the dependant variable.
(DOCX)

S2 Table EXPERIMENT 1. Multiple linear regression coefficients with each subscale of the questionnaires as the independent variables and the “coercion effect” of commanders as the dependant variable.
(DOCX)

S3 Table EXPERIMENT 1. Multiple linear regression coefficients with each subscale of the questionnaires as the independent variables and the “direct agency effect” as the dependant variable.
(DOCX)

S4 Table Multiple linear regression coefficients with each subscale of the questionnaires as the independent variables and the “agent’s coercion effect” as the dependant variable. (DOCX)

S5 Table Section a) EXPERIMENT 2. Multiple linear regression coefficients with each subscale of the questionnaires as the independent variables and antisocial disobedience as the dependant variable. Section b) EXPERIMENT 2. Multiple linear regression coefficients with each subscale of the questionnaires as the independent variables and prosocial disobedience as the dependant variable.
(DOCX)

S6 Table EXPERIMENT 2. Multiple linear regression coefficients with each subscale of the questionnaires as the independent variables and the “corrected commander’s coercion effect” as the dependant variable.
(DOCX)

S1 Fig Graphical representation of the agents’ and commanders’ corrected interval estimates. All tests were two-tailed. Errors bars represent standard errors. *** indicates a p value < = .001.
(DOCX)

S2 Fig Disobedience rates. Percentage of trials in which agents decided to disobey in the free-choice condition, either to administer a shock while saying not to (antisocial disobedience) or not to administer a shock while saying to (prosocial disobedience). Test was two-tailed. Errors bars represent standard errors.
(DOCX)
