# Supplementary material for: Only giving orders? An experimental study of the sense of agency when giving or receiving commands
Source: PLoS One. 2018 Sep 26;13(9):e0204027. doi: 10.1371/journal.pone.0204027 (PMC6157880; doi:10.1371/journal.pone.0204027)
Supplement: S1 Text — (DOCX) [file pone.0204027.s001.docx]

**S1 Text. Debriefing mail**

Dear NAME,

A few weeks ago, you have participated in an experiment designed to study the relationship between the sense of agency and the feeling of responsibility in a situation of coercion. You participated in this study with two other volunteers, and played three roles: ‘commander’, ‘agent’, and ‘victim’. To remember, when you were in the role of the agent, you could either decide to inflict or not an electric shock to the ‘victim’ in order to earn + €0.05, or you were asked to follow the orders of the commander. When you were in the role of the commander, you could either freely decide to give the order to the agent to inflict or not a shock to the ‘victim’ in order to earn + €0.05, or you had no decisional power on the agent’s action.

We are currently conducting a debriefing about what you felt during and after this experiment. It is possible to make an appointment by phone in order to answer to the different questions. Nonetheless, if you prefer answering by mail, you can send us back your answers.

1. What did you feel when you were in the role of the commander?
2. What did you feel when you were in the role of the agent?
3. What did you feel when you were in the role of the victim?
4. Do you have any other comments about the experiment?

Thank you in advance for your participation,

Best regards,

Emilie Caspar
