# Supplementary material for: Only giving orders? An experimental study of the sense of agency when giving or receiving commands
Source: PLoS One. 2018 Sep 26;13(9):e0204027. doi: 10.1371/journal.pone.0204027 (PMC6157880; doi:10.1371/journal.pone.0204027)
Supplement: S2 Text — Section a) EXPERIMENT 1. Repeated-measures ANOVA with Role (Agent, Commander), Condition (Free-choice, Coercive), Outcome (Shock, No shock) as within-subject factors and Order of the role as a between-subjects factor on corrected interval estimates. Section b) EXPERIMENT 1. Repeated-measures ANOVA with Role (Agent, Commander), Condition (Free-choice, Coercive), Outcome (Shock, No shock) and Delay (200, 500, 800 ms) as within-subject factors and Order of the role as a between-subjects factor on corrected interval estimates. (DOCX) [file pone.0204027.s002.docx]

**S2 Text. Section a) EXPERIMENT 1. Repeated-measures ANOVA with Role (Agent, Commander), Condition (Free-choice, Coercive), Outcome (Shock, No shock) as within-subject factors and Order of the role as a between-subjects factor on corrected interval estimates.**

The main effect of Role was not significant (*p* > .8), suggesting that interval estimates did not differ when participants were agents or commanders. The main effect of Condition was significant (F(1,29)=16.195, *p* < .001, η^2^***_partial_*** = .358), with coercion leading to longer corrected interval estimates (implying less sense of agency) than free choice (-7.5 ms, CI_95_=(-)41-26 and -34.5 ms, CI_95_= (-)68-.1), suggesting again that coercion reduces the implicit feeling of agency, as measured through this method. The interaction Role x Condition was significant (F(1,29)=5.66, *p* = .025, η^2^***_partial_*** = .161). Paired comparisons indicated that interval estimates were shorter in the free-choice than in the coercive condition for agents (t(32)=-4.242, *p* < .001), but that this difference was not significant for commanders (*p* > .9), see **S1 Fig**. None other factors or interaction were significant (all *p*s > .3).

**S2 Text. Section b) EXPERIMENT 1. Repeated-measures ANOVA with Role (Agent, Commander), Condition (Free-choice, Coercive), Outcome (Shock, No shock) and Delay (200, 500, 800 ms) as within-subject factors and Order of the role as a between-subjects factor on corrected interval estimates.**

The only significant interaction was Role x Condition x Delay (F(2,50)=4.319, *p* = .019, η^2^*_partial_* = .147). To understand this interaction, we run the same analysis on agents’ interval estimates and on commanders’ interval estimates. When participants were in the role of commander, the interaction Condition x Delay was not significant (*p* > .5). It thus suggests that the lack of difference between conditions for commanders is not influenced by the factor delay. When participants were in the role of agent, the interaction Condition x Delay was significant (F(2,54)=7.278, *p* = .002, η^2^*_partial_* = .212). Paired comparisons indicated that the difference between the free-choice and the coercive conditions was significant for all three delays for agents (200ms: t(31)=-3.310, *p* = .002 – 500 ms: t(30)=-2.360, *p* = .025 – 800 ms: t(30)=-3.444, *p* = .002), always with shorter interval estimates in the free-choice than in the coercive condition. Further paired-comparisons indicated that the difference between the free-choice and the coercive condition was greater for the 800 ms delay than for the 200 ms delay (t(29)=-3.304, *p* = .003) and marginally greater than the 500 ms delay (t(30)=-1.943, *p* = .061). The difference between the 200 ms delay and the 500 ms delay did not differ (p > .8). It thus suggests that the modulation of the coercion effect was greater for long interval estimates than for short ones.
