# Supplementary material for: Only giving orders? An experimental study of the sense of agency when giving or receiving commands
Source: PLoS One. 2018 Sep 26;13(9):e0204027. doi: 10.1371/journal.pone.0204027 (PMC6157880; doi:10.1371/journal.pone.0204027)
Supplement: S3 Text — Disobedience rates. (DOCX) [file pone.0204027.s003.docx]

**S3 Text.** **EXPERIMENT 2. Disobedience rates**. Importantly, in the free-choice condition, agents were explicitly told that they could choose to disobey or not the commander’s instructions. Even though the possibility to disobey was explicitly offered to agents, they chose to disobey only in 32 % of trials (CI_95_= 26-38). Paired comparisons indicated that agents were statistically no more likely to disobey when ordered by the commander to administer a shock (18% ‘prosocial’ disobedience, CI_95_= 13-23) than when ordered not to administer a shock (14% ‘antisocial’ disobedience, CI_95_= 10-18), *p* > .3: the number of shocks that commanders instructed to deliver was included as a covariate in this analysis) (**S2 Fig**). This suggests that disobedience was not strongly related to empathetic concern for the “victim”. Section a in S5 Table and Section b in S5 Table report supplementary multiple linear regressions conducted to assess whether or not personality traits predicted antisocial and prosocial disobedience.
