# Supplementary material for: Only giving orders? An experimental study of the sense of agency when giving or receiving commands
Source: PLoS One. 2018 Sep 26;13(9):e0204027. doi: 10.1371/journal.pone.0204027 (PMC6157880; doi:10.1371/journal.pone.0204027)
Supplement: S1 Table — Multiple linear regression coefficients with each subscale of the questionnaires as the independent variables and the “coercion effect” of agents as the dependant variable. (DOCX) [file pone.0204027.s004.docx]

**S1 Table. EXPERIMENT 1. Multiple linear regression coefficients with each subscale of the questionnaires as the independent variables and the “coercion effect” of agents as the dependant variable.**

| Questionnaires | **Unstandardized coefficients** | | **Standardized coefficients** |
| --- | --- | --- | --- |
|  | Beta | Std. Error | Beta |
| (Constant) | -172.18 | 314.44 |  |
| **Social Dominance Orientation scale** | 41.141 | 74.99 | .134 |
| **Interpersonal Reactivity Index** |  |  |  |
| *IRI - Perspective taking* | 71.12 | 107.85 | .157 |
| *IRI - Fantasy* | 111.91 | 128.89 | .204 |
| *IRI - Empathic concern* | 12.62 | 130.52 | .023 |
| *IRI - Personal distress* | 71.80 | 70.76 | .213 |
| **Levenson Self-Report Psychopathy scale** |  |  |  |
| *LSRP – primary psychopathy* | -39.91 | 155.118 | -.061 |
| *LSRP – secondary psychopathy* | .855 | 145.16 | .001 |

***Interpersonal Reactivity Index***, *IRI*. *Perspective taking* = the tendency to spontaneously adopt the psychological point of view of others. *Fantasy* = taps respondents’ tendencies to transpose themselves imaginatively into the feelings and actions of fictitious characters in books, movies and plays. *Empathic concern* = assesses ‘other-oriented’ feelings of sympathy and concern for unfortunate others. *Personal distress* = measures ‘self-oriented’ feelings of personal anxiety and unease in tense interpersonal settings. ***Levenson Self-Report Psychopathy scale***, LRSP. *Primary psychopathy* = selfish, uncaring, and manipulative posture towards others. *Secondary psychopathy* = impulsivity and self-defeating lifestyle. ***Social Dominance Orientation scale*** = assesses one’s degree of preference for inequality among social groups.
