# Supplementary material for: Only giving orders? An experimental study of the sense of agency when giving or receiving commands
Source: PLoS One. 2018 Sep 26;13(9):e0204027. doi: 10.1371/journal.pone.0204027 (PMC6157880; doi:10.1371/journal.pone.0204027)
Supplement: S2 Table — Multiple linear regression coefficients with each subscale of the questionnaires as the independent variables and the “coercion effect” of commanders as the dependant variable. (DOCX) [file pone.0204027.s005.docx]

**S2 Table. EXPERIMENT 1. Multiple linear regression coefficients with each subscale of the questionnaires as the independent variables and the “coercion effect” of commanders as the dependant variable.**

| Questionnaires | **Unstandardized coefficients** | | **Standardized coefficients** |
| --- | --- | --- | --- |
|  | Beta | Std. Error | Beta |
| (Constant) | -375.81 | 325.82 |  |
| **Social Dominance Orientation scale** | 14.25 | 77.70 | .040 |
| **Interpersonal Reactivity Index** |  |  |  |
| *IRI - Perspective taking* | -159.56 | 111.75 | -.307 |
| *IRI - Fantasy* | -59.04 | 133.55 | -.094 |
| *IRI - Empathic concern* | 256.50 | 135.24 | .415 |
| *IRI - Personal distress* | -78.90 | 73.32 | -.205 |
| **Levenson Self-Report Psychopathy scale** |  |  |  |
| *LSRP – primary psychopathy* | 269.98 | 160.73 | .363 |
| *LSRP – secondary psychopathy* | 137.55 | 150.41 | .182 |
