# Supplementary material for: Only giving orders? An experimental study of the sense of agency when giving or receiving commands
Source: PLoS One. 2018 Sep 26;13(9):e0204027. doi: 10.1371/journal.pone.0204027 (PMC6157880; doi:10.1371/journal.pone.0204027)
Supplement: S3 Table — Multiple linear regression coefficients with each subscale of the questionnaires as the independent variables and the “direct agency effect” as the dependant variable. (DOCX) [file pone.0204027.s006.docx]

**S3 Table. EXPERIMENT 1. Multiple linear regression coefficients with each subscale of the questionnaires as the independent variables and the “direct agency effect” as the dependant variable.**

| Questionnaires | **Unstandardized coefficients** | | **Standardized coefficients** |
| --- | --- | --- | --- |
|  | Beta | Std. Error | Beta |
| (Constant) | -1491.0 | 413.93 |  |
| **Social Dominance Orientation scale** | 181.0 | 98.72 | .341 |
| **Interpersonal Reactivity Index** |  |  |  |
| *IRI - Perspective taking* | -76.89 | 141.97 | -.098 |
| *IRI - Fantasy* | 146.22 | 169.67 | .155 |
| *IRI - Empathic concern* | 411.55 | 171.81 | .442 |
| *IRI - Personal distress* | 16.205 | 93.147 | .028 |
| **Levenson Self-Report Psychopathy scale** |  |  |  |
| *LSRP – primary psychopathy* | 320.64 | 204.19 | .286 |
| *LSRP – secondary psychopathy* | 585.45 | 191.08 | .513 |
