# Supplementary material for: Only giving orders? An experimental study of the sense of agency when giving or receiving commands
Source: PLoS One. 2018 Sep 26;13(9):e0204027. doi: 10.1371/journal.pone.0204027 (PMC6157880; doi:10.1371/journal.pone.0204027)
Supplement: S4 Table — (DOCX) [file pone.0204027.s007.docx]

**S4 Table. Multiple linear regression coefficients with each subscale of the questionnaires as the independent variables and the “agent’s coercion effect” as the dependant variable.**

| Questionnaires | **Unstandardized coefficients** | | **Standardized coefficients** |
| --- | --- | --- | --- |
|  | Beta | Std. Error | Beta |
| (Constant) | -163.17 | 461.50 |  |
| **Social Dominance Orientation scale** | 26.55 | 113.06 | .052 |
| **Interpersonal Reactivity Index** |  |  |  |
| *IRI - Perspective taking* | 111.28 | 216.73 | .140 |
| *IRI - Fantasy* | 20.45 | 181.63 | .024 |
| *IRI - Empathic concern* | 77.03 | 295.75 | .082 |
| *IRI - Personal distress* | -213.17 | 137.01 | -.420 |
| **Levenson Self-Report Psychopathy scale** |  |  |  |
| *LSRP – primary psychopathy* | 112.10 | 224.53 | .120 |
| *LSRP – secondary psychopathy* | 98.14 | 161.78 | .123 |
