# Supplementary material for: Only giving orders? An experimental study of the sense of agency when giving or receiving commands
Source: PLoS One. 2018 Sep 26;13(9):e0204027. doi: 10.1371/journal.pone.0204027 (PMC6157880; doi:10.1371/journal.pone.0204027)
Supplement: S5 Table — Section a) EXPERIMENT 2. Multiple linear regression coefficients with each subscale of the questionnaires as the independent variables and antisocial disobedience as the dependant variable. Section b) EXPERIMENT 2. Multiple linear regression coefficients with each subscale of the questionnaires as the independent variables and prosocial disobedience as the dependant variable. (DOCX) [file pone.0204027.s008.docx]

**S5 Table. Section a) EXPERIMENT 2. Multiple linear regression coefficients with each subscale of the questionnaires as the independent variables and antisocial disobedience as the dependant variable.**

| Questionnaires | **Unstandardized coefficients** | | **Standardized coefficients** |
| --- | --- | --- | --- |
|  | Beta | Std. Error | Beta |
| (Constant) | -8.19 | 27.20 |  |
| **Social Dominance Orientation scale** | 7.32 | 6.66 | .193 |
| **Interpersonal Reactivity Index** |  |  |  |
| *IRI - Perspective taking* | -35.89 | 12.77 | -.600 |
| *IRI - Fantasy* | 29.51 | 10.71 | .463 |
| *IRI - Empathic concern* | 14.38 | 17.435 | .204 |
| *IRI - Personal distress* | -11.57 | 8.07 | -.305 |
| **Levenson Self-Report Psychopathy scale** |  |  |  |
| *LSRP – primary psychopathy* | 46.33 | 13.23 | .664 |
| *LSRP – secondary psychopathy* | -24.10 | 9.54 | -.405 |

**S5 Table. Section b) EXPERIMENT 2. Multiple linear regression coefficients with each subscale of the questionnaires as the independent variables and prosocial disobedience as the dependant variable.**

| Questionnaires | **Unstandardized coefficients** | | **Standardized coefficients** |
| --- | --- | --- | --- |
|  | Beta | Std. Error | Beta |
| (Constant) | -20.42 | 30.17 |  |
| **Social Dominance Orientation scale** | 5.02 | 7.39 | .159 |
| **Interpersonal Reactivity Index** |  |  |  |
| *IRI - Perspective taking* | 8.36 | 14.16 | .168 |
| *IRI - Fantasy* | 1.044 | 11.87 | .020 |
| *IRI - Empathic concern* | 14.66 | 19.33 | .249 |
| *IRI - Personal distress* | -7.18 | 8.95 | -.227 |
| **Levenson Self-Report Psychopathy scale** |  |  |  |
| *LSRP – primary psychopathy* | 1.16 | 14.67 | .020 |
| *LSRP – secondary psychopathy* | 11.32 | 10.57 | .228 |
