# Supplementary material for: Only giving orders? An experimental study of the sense of agency when giving or receiving commands
Source: PLoS One. 2018 Sep 26;13(9):e0204027. doi: 10.1371/journal.pone.0204027 (PMC6157880; doi:10.1371/journal.pone.0204027)
Supplement: S6 Table — Multiple linear regression coefficients with each subscale of the questionnaires as the independent variables and the “corrected commander’s coercion effect” as the dependant variable. (DOCX) [file pone.0204027.s009.docx]

**S4 Table. EXPERIMENT 2. Multiple linear regression coefficients with each subscale of the questionnaires as the independent variables and the “corrected commander’s coercion effect” as the dependant variable.**

| Questionnaires | **Unstandardized coefficients** | | **Standardized coefficients** |
| --- | --- | --- | --- |
|  | Beta | Std. Error | Beta |
| (Constant) | -1335.25 | 2493.44 |  |
| **Social Dominance Orientation scale** | -643.41 | 610.87 | -.238 |
| **Interpersonal Reactivity Index** |  |  |  |
| *IRI - Perspective taking* | 2005.36 | 1170.94 | .469 |
| *IRI - Fantasy* | -1400.29 | 981.33 | -.307 |
| *IRI - Empathic concern* | 483.76 | 1597.89 | .096 |
| *IRI - Personal distress* | 137.96 | 740.25 | .051 |
| **Levenson Self-Report Psychopathy scale** |  |  |  |
| *LSRP – primary psychopathy* | -615.47 | 1213.09 | -.123 |
| *LSRP – secondary psychopathy* | 1139.32 | 874.10 | .268 |
