# Supplementary material for: Only giving orders? An experimental study of the sense of agency when giving or receiving commands
Source: PLoS One. 2018 Sep 26;13(9):e0204027. doi: 10.1371/journal.pone.0204027 (PMC6157880; doi:10.1371/journal.pone.0204027)
Supplement: S1 Fig — All tests were two-tailed. Errors bars represent standard errors. *** indicates a p value < = .001. (DOCX) [file pone.0204027.s010.docx]

**S1 Fig. Graphical representation of the agents’ and commanders’ corrected interval estimates.** All tests were two-tailed. Errors bars represent standard errors. *** indicates a p value <= .001.
