# Supplementary material for: Only giving orders? An experimental study of the sense of agency when giving or receiving commands
Source: PLoS One. 2018 Sep 26;13(9):e0204027. doi: 10.1371/journal.pone.0204027 (PMC6157880; doi:10.1371/journal.pone.0204027)
Supplement: S2 Fig — Percentage of trials in which agents decided to disobey in the free-choice condition, either to administer a shock while saying not to (antisocial disobedience) or not to administer a shock while saying to (prosocial disobedience). Test was two-tailed. Errors bars represent standard errors. (DOCX) [file pone.0204027.s011.docx]

**S2 Fig*.*** **Disobedience rates.** Percentage of trials in which agents decided to disobey in the free-choice condition, either to administer a shock while saying not to (antisocial disobedience) or not to administer a shock while saying to (prosocial disobedience). Test was two-tailed. Errors bars represent standard errors
